# Laccase Activity in Fungus *Cryphonectria parasitica* Is Affected by Growth Conditions and Fungal–Viral Genotypic Interactions

**DOI:** 10.3390/jof7110958

**Published:** 2021-11-11

**Authors:** Lucija Nuskern, Mirta Tkalec, Bruno Srezović, Marin Ježić, Martina Gačar, Mirna Ćurković-Perica

**Affiliations:** 1 Department of Biology, Division of Microbiology, Faculty of Science, University of Zagreb, Marulićev trg 9a, 10000 Zagreb, Croatia; lucija.nuskern@biol.pmf.hr (L.N.); bsrezovic@gmail.com (B.S.); marin.jezic@biol.pmf.hr (M.J.); martina.gacar@gmail.com (M.G.); 2 Department of Biology, Division of Botany, Faculty of Science, University of Zagreb, Rooseveltov trg 6, 10000 Zagreb, Croatia; mirta.tkalec@biol.pmf.hr

**Keywords:** chestnut blight, *Cryphonectria hypovirus 1*, virus effect

## Abstract

Laccase activity reduction in the chestnut blight fungus *Cryphonectria parasitica* usually accompanies the hypovirulence caused by the infection of fungus with *Cryphonectria hypovirus 1* (CHV1). However, the different methods utilized for assessing this phenomenon has produced varied and often conflicting results. Furthermore, the majority of experimental setups included only one prototypic system, further confounding the results. Considering the diversity of fungal isolates, viral strains, and variability of their effects on the phytopathogenic process observed in nature, our goal was to ascertain if laccase activity variability is affected by (1) different *C. parasitica* isolates infected with several CHV1 strains, and (2) growth conditions. We have demonstrated that some CHV1 strains, contrary to previous assumptions, increase the activity of *C. parasitica* laccases. The specific fungal isolates used in the experiments and culture conditions also affected the results. Furthermore, we showed that two commonly used laccase substrates, 2,2′-azino-bis(3-ethylbenzothiazoline-6-sulphonic acid) and 2,4-dimethoxyphenol, cannot be used interchangeably in *C. parasitica* laccase activity measurements. Our results illustrate the importance of conducting this type of study in experimental systems and culture conditions that resemble natural conditions as much as possible to be able to infer the most relevant conclusions applicable to natural populations.

## 1. Introduction

Enzymes that catalyze the oxidation of polyphenols, methoxy-substituted phenols, aromatic diamines, and a range of other compounds, called laccases (benzenediol: oxygen oxidoreductase, EC 1.10.3.2; LAC), are commonly found in plants and fungi. The primary role of fungal laccases is lignin degradation, but intracellular forms of these enzymes have been found in many fungal species and are presumed to take part in other cellular processes, like morphogenesis, pigment synthesis, fungal/plant interactions, stress defense, and fruiting body development [1,2]. They are typical for litter-decomposing, wood-decaying, and phytopathogenic fungi. A screening of 11 available fungal phytopathogen genomes revealed 101 putative laccase genes [3]. This redundancy demonstrates the importance of these enzymes in plant pathogenic fungi.

*Cryphonectria parasitica* is a known phytopathogenic fungus in which laccase activity has been linked with its pathogenic potential [4]. Infection of chestnuts with this fungus causes blight disease, a pandemic that has ravaged chestnut forests worldwide, making this ascomycete one of the top 100 most dangerous invasive species according to the Global Invasive Species Database [5]. This pathogen has been studied for a long time due to its devastating effects on the stands of American (*Castanea dentata*) and European chestnut (*Castanea sativa*), as well as due to an interesting phenomenon called hypovirulence, caused by an infection of the fungus with the mycovirus *Cryphonectria hypovirus 1* (CHV1). Hypovirulence is characterized by reduced growth, sexual and asexual reproductive ability, and virulence of the infected fungus, leading to its debilitation which in turn facilitates the recovery of the diseased chestnuts [6,7]. Thus, treatment of the bark cankers, caused by the virulent *C. parasitica*, with hypovirulent fungal strains, has long been used in the biological control of chestnut blight, as the transmission of the mycovirus CHV1 transforms the virulent fungus into a hypovirulent one [8]. Furthermore, CHV1 infection reduces the activities of fungal laccase [4], cutinase [9] and polygalacturonase [10], the fungal enzymes involved in plant cell wall degradation and subsequently in chestnut blight.

Out of the 16 putative laccase genes found by analyzing the *C. parasitica* genome [3], three have been experimentally isolated and characterized: extracellular LAC1 [11,12], intracellular LAC2 [13] and extracellular, tannic acid-inducible LAC3 [14,15]. Activities of all three of the aforementioned LAC enzymes are usually suppressed upon CHV1 infection [11,13,14]. Nevertheless, results regarding CHV1′s effect on laccases in *C. parasitica* are often conflicting, especially when different methods and/or experimental designs are utilized. Activity of LAC1 and *lac1* mRNA accumulation were reduced when grown in liquid culture [11,13], but transcriptomic microarray analysis of cultures grown on solid medium with cellophane overlay showed that *lac1* transcript levels remained unchanged upon CHV1 infection [16]. On the other hand, analysis of *C. parasitica* secretome showed a reduction of LAC1 accumulation and an increase of the corresponding mRNA in CHV1-infected strains. The mycelia were grown in liquid culture and the aforementioned effect depended on the particular media used [17]. It is also worth noting that the majority of studies on CHV1′s effect on *C. parasitica* laccases was done using only one particular experimental system, that is, fungal isolate EP155 and its isogenic virus-infected strain EP155/EP713. Having in mind the vast diversity of fungal isolates and virus strains and their effects on the phytopathogenic processes observed in nature [18], it is important to investigate the effect of different virus strains on the fungus, especially when the assumed important role of laccases in the pathogenesis of chestnut blight disease is considered [15].

Therefore, the aims of this study were: (1) to determine the effect of different strains of CHV1 on intracellular and extracellular laccase activities in different *C. parasitica* isolates; (2) to assess the role of different culturing media on laccase activities; and (3) to evaluate two commonly used laccase substrates for use in *C. parasitica* laccase activity measurements.

## 2. Materials and Methods

### 2.1. Chemicals

Difco^TM^ potato dextrose agar (PDA), Difco^TM^ potato dextrose broth (PDB), and Bacto^TM^ malt extract (ME) media were purchased from Becton, Dickinson and Company (Franklin Lakes, NJ, USA); agar from Biolife (Milan, Italy); Comassie Brilliant Blue G 250, bovine serum albumin, 2,2′-azino-bis(3-ethylbenzothiazoline-6-sulphonic acid) (ABTS), 2,4-dimethoxyphenol (DMOP) and sodium acetate from Sigma-Aldrich (St. Louis, MI, USA); potassium dihydrogen phosphate and potassium hydrogen phosphate from Lach-Ner (Brno, Czech Republic); ethylenediaminetetraacetic acid (EDTA), phosphoric acid, disodium hydrogen phosphate (Na_2_HPO_4_) and 96% ethanol from Kemika (Zagreb, Croatia); acetic acid from J. T. Baker Chemicals (Radnor, PA, USA); citric acid from Gram-mol (Zagreb, Croatia). All chemicals were of the highest analytical grade.

### 2.2. Cryphonectria parasitica Isolates and CHV1 Strains

In this study, we used three virulent (VIR) isolates of *C. parasitica*: L14, D7 and D9 of the same vegetative compatibility (vc) type (EU-1) [19]. To convert these isolates into hypovirulent (HV) ones by hyphal anastomosis, we used CHV1-infected fungal isolates HK27 and CR23 (Croatian field isolates), SHE30 (Georgian field isolate) and prototypic EP713, as previously described [20]. In short, ~5 mm agar blocks of virus–donor and virus–recipient isolates were placed 1–2 mm apart on PDA in a 90 mm Petri plate. The plates were incubated in a climate chamber at 24 °C and 70% relative humidity for 7 days in dark followed by benchtop incubation for 7 days at room temperature. Confirmation of virus transfer was done by dsRNA extraction (Double-RNA Viral dsRNA extraction mini kit, iNtRON Biotechnology Inc., Seongnam, South Korea) and RT-PCR. First, strand cDNA synthesis was conducted using the GoScript™ Reverse Transcription System (Promega, Madison, WI, USA), using up to 5 μg of isolated dsRNA. The PCR reaction was performed for ORFA (primer pairs EP713-5 and R2280) and ORF B (primer pairs EP713-6 and EP713-7), as described by Allemann et al. [21]. Cycling parameters were 2 min at 94 °C followed by 35 cycles of 1 min at 94 °C, 1:30 min at 55 °C, 2 min at 72 °C, and a final extension of 10 min at 72 °C. A successful transfer of the virus was confirmed by observing amplicons of appropriate size after the agarose electrophoresis of the PCR products.

The CHV1 strains harbored in *C. parasitica* isolates HK27 and CR23 were previously determined to be of Italian subtype I [22], while the strain infecting isolate SHE30 was of French subtype F2/Georgian subtype G [23,24]. From here on, the CHV1 strains used in our experiments will be referred to with the designation of the fungal isolate from which they were obtained (e.g., HK27 will represent the CHV1 strain obtained from HK27 hypovirulent fungal isolate, thus L14/HK27 means fungal isolate L14 transfected with CHV1 from the HK27 isolate). A prototypic, virulent isolate EP155 (vc type EU-5) and its isogenic, hypovirulent counterpart, isolate EP713 were used as well [25]. The CHV1 strain in isolate EP713 belongs to the French subtype F1 [21]. No other virus strains were transferred to EP155, as we used this prototypic fungus/virus combination only as a control, considering its widespread use in *C. parasitica* laccase activity research [11,12,13,15,26]. All *C. parasitica* isolates were kept at −80 °C in 22% glycerol, and re-cultured on PDA prior to the experimental setup. After thawing the fungal cultures, the presence/absence of viral dsRNA was confirmed as described previously [20]. In total, 17 experimental cultures were obtained: four VIR fungal isolates: EP155, L14, D7, and D9; and 12 HV cultures designated as the fungal isolate/virus strain and HV fungal isolate EP713 (Table 1).

### 2.3. Cultivation of Experimental Cultures

To measure the activity of fungal laccases, we used four different growth media—two solid media: (1A) PDA and (1B) malt extract agar (MEA), and two liquid media: (2A) potato dextrose broth (PDB) and (2B) malt extract (ME). For all of the treatments, each sample was inoculated in triplicate.

#### 2.3.1. Cultivation on Solid Media

For cultivation on solid media (1A and 1B), the following method was used: a (2 × 2 × 2) mm agar block from an actively growing colony margin of three-day-old PDA culture was placed on a fresh 90 mm Petri plate containing 20 mL PDA or MEA, overlaid with sterile cellophane (Cellophane Membrane Backing, Bio-Rad, Hercules, California, USA). Samples were incubated in a climate chamber for either 5 (5D) or 10 (10D) days, in the dark at 24 °C and 70% relative humidity. After 5 or 10 days of growth, respectively, mycelia were stripped from the cellophane, transferred to previously weighted 2 mL tubes, and lyophilized.

#### 2.3.2. Cultivation on Liquid Media

For cultivation in liquid media (2A and 2B), the following method of cultivation was applied: five-day-old cultures grown on PDA were used, and the inoculum was obtained by flooding the colonies with 1 mL of sterile deionized water and gently scraping the surface with a sterile scalpel. The resulting suspension was transferred into tubes containing 10 mL of sterile PDB or ME medium and incubated in a climate chamber for 5D or 10D, in the dark at 24 °C and 70% relative humidity. After incubation, the mycelium was separated from the medium by decanting the liquid and transferring the mycelium into previously weighted 2 mL tubes. The separated mycelia were centrifuged at 25,000× *g* for 10 min at 4 °C to remove excess liquid and then washed twice with 1 mL of sterile deionized water, followed by centrifugation at 25,000× *g* for 5 min at 4 °C. After the final centrifugation, the water was decanted and the samples were quickly frozen at −80 °C and lyophilized. For measurement of extracellular laccase activity (ecLAC), the decanted media were stored at −20 °C.

In all cases, lyophilized tissue was ground to a fine powder with a steel ball in a TissueLyser II (Qiagen, Venlo, Netherlands) for 2 min at 30 Hz and the tubes were weighed again to determine the dry biomass used for LAC activity calculation. Ground tissue was stored at −20 °C until measurement.

### 2.4. Preparation of Protein Extracts

To prepare the protein extracts for activity assays, a cold extraction buffer (100 mM potassium phosphate buffer, with 0.1 mM EDTA, pH 7.0) was added to the ground lyophilized tissue, mixed with micro pestle and vortexed briefly. The mycelia obtained from 5D MEA were extracted with 0.5 mL of buffer, 5D/10D PDB, 5D/10D ME and 10D MEA with 1.0 mL and 5D/10D PDA with 1.5 mL, due to a different amount of collected tissue and in accordance with our preliminary assessment. The homogenate was centrifuged at 20,000× *g* for 20 min at 4 °C. Supernatant was collected and protein concentration was determined according to Bradford [27] using bovine serum albumin as a standard. All spectrophotometric measurements were done with a Specord 40 spectrophotometer (Analytik Jena, Jena, Germany).

### 2.5. Laccase Activity Assays

LAC activity was assayed spectrophotometrically using two different, commonly used laccase substrates: 2,2′-azino-bis(3-ethylbenzothiazoline-6-sulphonic acid) (ABTS) [28] and 2,4-dimethoxyphenol (DMOP) combining the methods described by Rigling et al. [4] and Palmieri et al. [29]. In all measurements, plastic 1 mL cuvettes were used.

To test the permeability of cellophane for extracellular laccase enzyme, all cultures were grown for 10 days on PDA supplemented with 0.2 g/L ABTS (PDA + ABTS as described in [30]) with and without cellophane overlay, respectively. The cultures were assessed daily for the presence of blue coloration, indicating laccase activity. An almost complete absence of blue coloration on cellophane-overlaid plates strongly indicated that extracellular laccase did not diffuse efficiently through the cellophane and that it accumulated on the top side of the cellophane. Thus, the laccase activity obtained in further experiments on solid media (PDA or MEA) was designated as solid media LAC (smLAC) and was considered to be the sum of all intracellular and most extracellular laccase activity.

For measuring smLAC and intracellular LAC (icLAC) activity utilizing ABTS as a substrate, 850 µL of 100 mM sodium acetate buffer (pH 3.5) was mixed with 50 µL of 50 mM ABTS (prepared in the same sodium acetate buffer; final concentration 2.5 mM) to which 100 µL of protein extract was added. LAC activity was measured as an increase in absorbance at 418 nm due to the generation of cationic radical ABTS•^+^ (ε = 36 mM^−1^ cm^−1^) and expressed in nmol of generated ABTS•^+^ per minute per mg of dry weight (dw).

For measuring smLAC and icLAC activity utilizing DMOP as a substrate, 800 µL of citrate-phosphate buffer (0.1 M Na_2_HPO_4_ and 0.05 M citric acid, pH 3.4) was mixed with 50 µL of 50 mM DMOP (prepared in the same citrate-phosphate buffer; final concentration 2.5 mM) to which 150 µL of protein extract was added and incubated at room temperature for 10 min. LAC activity was measured as an increase in absorbance at 468 nm due to DMOP oxidation to 3,3′,5,5′-tetramethoxydiphenylquinone (DMOPox, ε = 14.8 mM^−1^ cm^−1^) and expressed in nmol of the generated DMOPox per minute per mg of dry weight (dw).

For measuring ecLAC activity with either ABTS or DMOP as substrates, 850 µL of leftover liquid medium used for fungal growth (PDB or ME) was mixed with 150 µL of 50 mM ABTS or DMOP (prepared as above; final concentration 7.5 mM) and incubated at room temperature for 10 min. After that LAC activity was measured as described previously and expressed in nmol of generated ABTS•^+^ or DMOPox per minute per mg of dry weight of the corresponding mycelium (dw).

### 2.6. Data Analysis

To determine the effect of the substrate used in the LAC assay (i.e., differences in obtained measurements with ABTS or DMOP as a substrate), all data were analyzed with one-way ANOVA, separately for smLAC, icLAC, and ecLAC. Furthermore, the correlation between ABTS and DMOP enzyme activity data was determined for smLAC, icLAC, and ecLAC, and expressed as a Pearson correlation coefficient (r). All other factors with a potential effect on LAC activity (i.e., fungal isolate, virus strain, growth medium, duration of cultivation) were assessed with a main effects ANOVA using only DMOP data for comparability with previous *C. parasitica* research. The effect of the cultivation duration was determined for each growth medium separately by individual t-tests, while a comparison between growth media and cultivation duration was obtained with one-way ANOVA and post hoc Tukey honest significant difference (HSD) test. The difference in LAC activity between VIR fungal isolates, as well as the effect of different strains of CHV1 were assessed with one-way ANOVA and a post hoc Tukey HSD test using data obtained after 10D of growth utilizing DMOP as a substrate for LAC activity measurement.

The virus effect (VE) on LAC activity was calculated as the difference between each of the triplicate measurements of HV isolates and the mean of the corresponding isogenic VIR isolate, and was given as a percentage (%) of the performances of the VIR isolate:

(1) $$\mathrm{VE}=\frac{\mathrm{LAC}\;\left(\mathrm{HV}\right)-\;\mathrm{MEAN}\;\left(\mathrm{LAC}\;\left(\mathrm{VIR}\right)\right)}{\mathrm{MEAN}\;\left(\mathrm{LAC}\;\left(\mathrm{VIR}\right)\right)}*100$$

A negative VE value indicated a higher level of LAC activity of VIR isolates, whereas a positive value showed a higher level of LAC activity of HV isolates. To determine the responsiveness of each fungal isolate to different virus strains, we tested the statistical significance of VE separately for each fungal isolate with one-way ANOVA and a post hoc Tukey HSD test. The data were expressed as means of three replicates with the corresponding standard errors.

Statistical analyses were done with Statistica 13 (StatSoft Inc., Tulsa, Oklahoma, USA). All statistical tests were done at *p* < 0.05.

## 3. Results

Laccase activity assays were performed with two different, commonly used, substrate chemicals, ABTS and DMOP. Analysis of results with one-way ANOVA showed a statistically significant difference between the results obtained with these two methods for smLAC (mean(ABTS) = 1.180049 ± 1.490070; mean(DMOP) = 2.849902 ± 4.633117; sum of squares (SS) = 284.18; degrees of freedom (df) = 1; mean square value (MS) = 284.418; F ratio (F) = 24.0156; *p* = 0.000001), icLAC (mean(ABTS) = 2.018775 ± 1.021927; mean(DMOP) = 0.755000 ± 0.937314; SS = MS = 162.9069; df = 1; F = 169.4393; *p* = 0.00) and ecLAC (mean(ABTS) = 0.550441 ± 0.920169; mean(DMOP) = 0.184069 ± 0.385081; SS = MS = 13.6913; df = 1; F = 27.5203; *p* = 0.000000). On solid media, there was a strong significant positive correlation between ABTS and DMOP data (r = 0.858782, *p* < 0.05). However, on liquid media, the correlation between the two methods was not significant (r(icLAC) = −0.248570, *p* > 0.05; r(ecLAC) = 0.071578, *p* > 0.05). Due to the lack of correlation between the methods, and because, to the best of our knowledge, all previous *C. parasitica* laccase activity research was done with DMOP as the substrate, our further analyses included only the DMOP data, so that we would be able to compare our results with the ones found in the relevant literature.

We wanted to assess the effect of growth conditions (medium solidity, duration of cultivation, and medium composition) and the effect of both the host and the virus’ genetic variability on *C. parasitica* LAC activity. As expected, the effects on fungal growth itself were also observed. For all media, the *t*-test showed a significant difference in dry weight between samples grown for 5D and 10D (*p* ≤ 0.0001). Moreover, the cultures grown on nutritiously poor ME and MEA had a fragile-looking colony morphology and grew slowly. The solidity of the growth medium (i.e., solid or liquid) also showed a marked impact on fungal growth. The highest biomass production was observed when samples were grown for 10D on PDA (average obtained dry weight, 77.3 mg), which differed significantly (*p* < 0.0001) from all other media at 10D of cultivation (average obtained dry weights: PDB = 19.9 mg, MEA = 9.7 mg, ME = 8.1 mg). When we compared the values of LAC activity of fungi grown on solid media (smLAC) with the total LAC values obtained from fungi in liquid media (sum of icLAC and ecLAC), the results showed that the effect of growth medium solidity was significant, with *p* < 0.0001. Because of this, all subsequent analyses were done separately for cultures grown on solid media and separately for the ones grown in liquid media.

For solid media laccase activity (smLAC), the main effects ANOVA showed that it was significantly affected by the duration of cultivation, growth medium composition, particular fungal isolate, and virus strain (Table 2). On liquid media, intracellular laccase activity (icLAC) was also significantly affected by the duration of cultivation, growth medium composition, fungal isolate, and virus strain, while extracellular laccase activity (ecLAC) was only affected by the virus strain. Since we observed that in some samples, the detectable LAC activity appeared only after 10 days of cultivation (data not shown), we chose a 10D, DMOP measured dataset as the relevant one for further analyses.

The laccase activities differed among uninfected (VIR) fungal isolates and were impacted by the growth medium (Table 3). For cultures grown on MEA (smLAC) or on ME (icLAC), there was no variability in laccase activity between fungal isolates. However, when grown on PDB, isolate L14 had a significantly higher ecLAC activity than the other three isolates. Furthermore, fungal isolates EP155 and D9 grown on ME had low ecLAC activity, while for isolates L14 and D7, ecLAC was not measurable at all.

**Figure 1:**
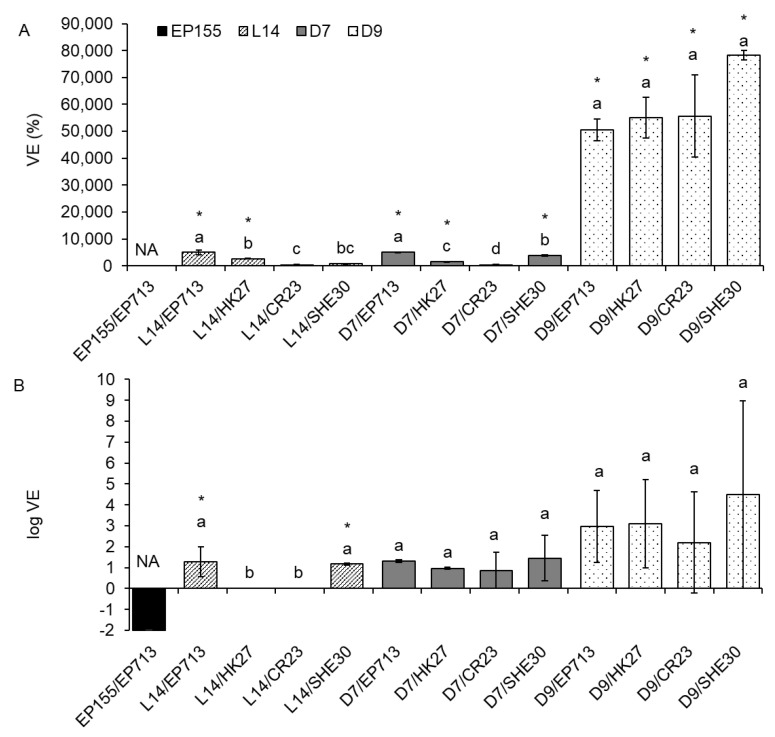
Effect of virus infection on solid media laccase (smLAC) activity of four *Cryphonectria parasitica* isolates when grown on (**A**) potato dextrose agar and (**B**) malt extract agar. Results in (**A**) are represented as mean virus effect (VE) value (difference between each triplicate measurement of HV isolate LAC activity and the mean of the corresponding isogenic VIR isolate LAC activity, given as a percentage of the performances of the VIR isolate) ± standard error. Results in (**B**) are in logarithmic scale. An asterisk (*) denotes virus-infected cultures with smLAC values significantly different from the corresponding VIR fungal isolate. Letters denote statistical differences between different virus strains infecting the same fungal isolate at *p* < 0.05. NA = not applicable to the statistical comparison of virus strains due to only one fungus/virus combination. The VE in EP155/EP713 (**A**) and L14/HK27 and L14/CR23 (**B**) amounted to zero, as in both VIR and HV fungi the smLAC activity was zero.

Furthermore, we wanted to define the particular effect of various virus strains on the LAC activity of the infected fungus. On solid PDA medium, CHV1 infection mostly led to an increase in laccase activity, as seen from the determined positive VE values (Figure 1). The increase was significant in most (9 out of 13) of the infected isolates in comparison to the VIR control. On MEA, an increase in laccase activity was usually observed and was significant in 2 out of 13 cases (columns marked with an asterisk in Figure 1), but in two samples, the VE equaled zero, as both the VIR and the HV sample had no measurable smLAC activity. In the prototypic EP155/EP713 experimental system grown on PDA (Figure 1A), there was no observed change upon CHV1 infection, as in both EP155 and EP155/EP713 the smLAC activity equaled zero. On the other hand, in EP155 and EP155/EP713 grown on MEA (Figure 1B), CHV1-infected mycelium showed a 100% reduction of smLAC activity. On PDA, virus strain EP713 had the strongest positive VE in fungal isolates L14 and D7. All of the tested viral isolates had a different VE in fungal isolate D7, while in fungal isolate L14, three out of four viruses exhibited a statistically different effect (Figure 1A). On the other hand, in fungal isolate D9, all CHV1 strains had a similar, very strong, positive VE. On MEA, no difference in VE was seen among virus strains, although D9/SHE30 had a VE that was two to three orders of magnitude higher than the other samples, but with extreme variance (Figure 1B, results are presented in logarithmic scale due to extreme values).

**Figure 2:**
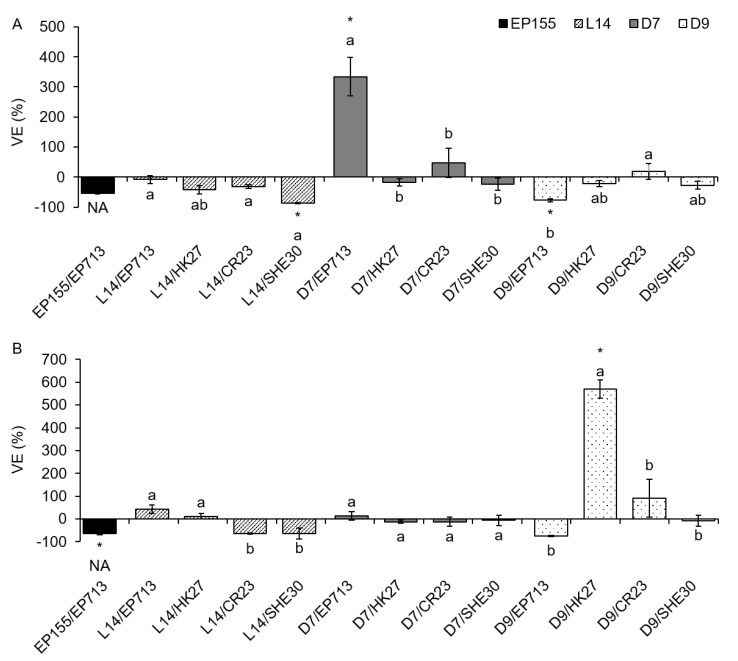
Effect of virus infection on the intracellular laccase (icLAC) activity of four *Cryphonectria parasitica* isolates when grown on liquid media (**A**) potato dextrose broth and (**B**) malt extract. Results are given as mean virus effect (VE) value (difference between each triplicate measurement of HV isolate LAC activity and the mean of the corresponding isogenic VIR isolate LAC activity, given as a percentage of the performances of the VIR isolate) ± standard error. An asterisk (*) denotes virus-infected cultures with icLAC values significantly different from the corresponding VIR fungal isolate. Letters denote statistical differences between different virus strains infecting the same fungal isolate at *p* < 0.05. NA = not applicable to the statistical comparison of virus strains due to only one fungus/virus combination.

On liquid media (PDB and ME), we analyzed VE separately for intra- and extracellular laccase activity, and the obtained results varied more than on solid media. On both media, the VE for icLAC showed both positive and negative values (i.e., increase and decrease of LAC activity upon CHV1 infection) although those deviations from VIR fungi were significant only in a few cases (Figure 2). Unlike the results obtained on solid media, some of those significant VE values were negative (L14/SHE30 and D9/EP713 on PDB; EP155/EP713 on ME), while others were positive (D7/EP713 on PDB; D9/HK27 on ME).

**Figure 3:**
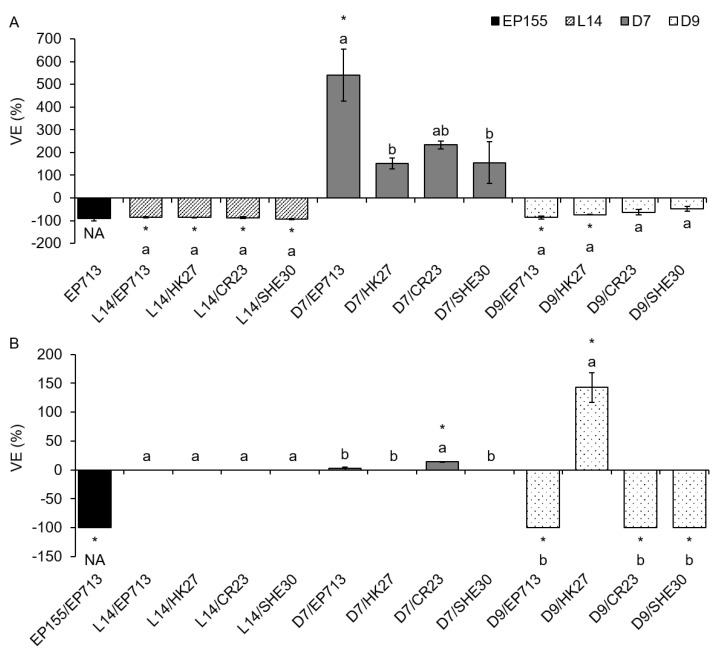
Effect of virus infection on extracellular laccase (ecLAC) activity of four *Cryphonectria parasitica* isolates when grown on liquid media (**A**) potato dextrose broth and (**B**) malt extract. Results are represented as mean virus effect (VE) value (difference between each triplicate measurement of HV isolat’ LAC activity and the mean of the corresponding isogenic VIR isolate LAC activity, given as a percentage of the performances of the VIR isolate) ± standard error. An asterisk (*) denotes virus-infected cultures with ecLAC values significantly different from the corresponding VIR fungal isolate. Letters denote statistical differences between different virus strains infecting the same fungal isolate at *p* < 0.05. NA = not applicable to the statistical comparison of virus strains due to only one fungus/virus combination. VE in (**B**) for all L14 isolates, D7/HK27 and D7/SHE30 equaled zero, as in both VIR and HV fungi the smLAC activity equaled zero.

Upon CHV1 infection, ecLAC activity showed to be more responsive than icLAC. More precisely, on PDB medium, all 13 VE values exhibited change when compared to VIR controls, and 7 out of those 13 changes were significantly different. On ME, although only 7 out of 13 samples showed any VE, 6 out of these 7 values were significantly different compared to VIR controls (Figure 3A). The majority of fungus/virus combinations showed reduced ecLAC activity upon infection, but in some cases, increased ecLAC activity was observed. The most profound differences appeared to be a consequence of the particular fungal isolate genotype. For example, infection of the isolate L14 with either one of the four studied viruses completely suppressed ecLAC activity when grown on PDB. The same fungal isolate when grown on ME showed no LAC activity, regardless of infection with either of the studied viruses, which is why the VE could not be calculated (Figure 3B). On the other hand, in isolate D7, VE was positive on both media, but only infection with CHV1 strain EP713 on PDB and CR23 on ME significantly increased laccase activity. The laccase activity of isolate D9 decreased upon CHV1 infection when grown on either PDB or ME, except for the viral strain HK27 which significantly increased ecLAC activity on ME.

## 4. Discussion

Various phenolic compounds can be oxidized by fungal laccases, so several substrates have been commonly used to assess their activities, with guaiacol, syringaldazine, ABTS, and DMOP being the most common ones [31]. Surprisingly, our experiments revealed significant differences in LAC activities when measured utilizing DMOP and ABTS assays. Specifically, on solid media the activities obtained by the two methods showed a strong positive correlation. However, on liquid media, no such correlation was observed for either extra- or intracellular laccase activity. The observed effect could be explained by the fact that DMOP is a phenolic compound, while ABTS is not. ABTS is also a substrate for peroxidases [32,33] so it might have been partly oxidized and therefore less available for the laccases. The large variability of laccase activity obtained in liquid media may indicate a lesser stability of this system compared to solid media, which could explain the lack of correlation between data. Laccase activity measurements of *C. parasitica* are commonly performed with DMOP as substrate, hence we primarily focused on the data obtained by DMOP assay.

The reduced laccase activity of hypovirulent *C. parasitica* isolates was one of the first biochemical traits directly attributed to CHV1 infections [4]. This first study was done on three virulent and three hypovirulent *C. parasitica* isolates from Switzerland. All of the virulent isolates showed strong laccase activity, while in one hypovirulent isolate, laccase activity was reduced by half and undetectable in the rest. With the exception of the study by Rigling et al. [4], other specific research on laccases of *C. parasitica* (i.e., regulation of biosynthesis, enzymes purification and molecular characterization, regulation of transcription) was done on the prototypic experimental system EP155/EP713 [11,12,13,14,15,26], where virulent fungal isolate EP155 originating from the United States was transfected with the European CHV1 strain. Considering our previous results regarding effects of a CHV1 infection on the stress enzyme activity [20] and epigenetic changes in the *C. parasitica* genome [34], which showed great variability between fungal isolates and fungus/virus combinations, we found it necessary to verify if indeed all of the virus strains in the different fungal isolates and combinations had the same suppressive effect on the laccase activity of *C. parasitica*. Furthermore, having in mind previous misconceptions regarding reduced oxalate synthesis in hypovirulent *C. parasitica* [35], which was actually the effect of applying a different nutritive medium [36], we wanted to assess the effect of different growth conditions as well (i.e., nutritive medium and duration of cultivation) to confirm the observed effects. The results presented in this paper clearly show that some CHV1 strains, in contrast to previous knowledge, increase the activity of *C. parasitica* laccases, and that the effect is strongly influenced by culture conditions, as well as fungal/viral genotype.

The significant effect of growth medium on laccase activity was to be expected. For example, fungi cultivated on either solid or in liquid media can exhibit morphological and physiological differences [37]. As higher fungi evolved primarily as terrestrial organisms, they are well adapted to grow on solid substrates, hence cultivation in liquid culture may change their metabolic response [38]. For instance, solid state-related gene expression induction was observed for solid state fermentation-specific glucoamylase gene *glaB* of *Aspergillus oryzae* grown on a nylon membrane placed over an agar plate medium [39]. Although we have not observed a similar induction, the physiological differences of fungi cultured on solid or in liquid media might have affected the biosynthesis of laccases and the subsequent differences in their enzymatic activities observed in this experiment. Wang et al. [17] analyzed the secretome of hypovirulent (CHV1-infected) *C. parasitica* isolates and showed an increased expression of *lac-1* gene in cultures grown on PDA but a decreased secretion of LAC enzyme in CHV1-infected cultures grown on EP complete liquid medium. On the other hand, a transcriptomic study by Allen et al. [16] showed no change in *lac-1* transcription for CHV1-infected *C. parasitica* grown on cellophane-overlaid PDA, thus making it difficult to infer any solid conclusion regarding *C. parasitica* laccase physiology in solid/liquid media. Our findings partially support the findings by Wang et al. [17], as we observed that CHV1 infection significantly increased smLAC activity in most samples grown on PDA and in two samples grown on MEA, and it decreased ecLAC activity in the majority of samples on PDB and ME (where VE ≠ 0). This indicates that in our case, the impact of the solidity of the medium (solid vs. liquid) cannot be disregarded.

Another important aspect of growth medium that can significantly influence the physiology of the fungus is its nutritive composition and duration of cultivation [31]. We observed a fragile culture morphology and poor biomass production of the cultures grown on nutritiously poor ME and MEA. Additionally, the difference in dry weight between cultures grown for either five or ten days correlated well with the observation that in some cultures measurable laccase activity was not observed after 5 days of growth and justified our need to prolong the cultivation to 10 days. The culture medium and age of the culture were reported previously to affect *C. parasitica* laccase mRNA accumulation [12]. The dependence of the variability in laccase biosynthesis on culture media was also observed in *Botrytis cinerea* [40]. Another study on *C. parasitica* showed repressed *lac1* transcription in rich medium (PDB), which could be relieved by transferring the culture from PDB to ME [26]. Considering that ME is a poor source of nitrogen and limited in amino acids, it was suggested that *lac1* transcription was regulated by the availability of nutrients, especially amino acids. In our study, virus-free isolates did not exhibit consistently higher LAC activity values (Table 3) in amino acid-poor media (MEA and ME) compared to rich media (PDA or PDB) that would go in line with the previously reported *lac1* transcription induction [26]. Instead, significantly lower ecLAC activities were observed in our study when *C. parasitica* was grown in poor rather than rich media. Considering the slightly different experimental setup, and a different timepoint for sample collection (hours in [26] vs. days in our experiment) it could be argued that the regulation, synthesis and secretion of *C. parasitica* laccases is a highly dynamic process sensitive to many parameters, as shown previously for other fungi [41].

In prototypic experimental system EP155/EP713, we observed a reduction in LAC activity in most cases except on PDA, where a discernable effect of this virus strain was not observed. Nevertheless, as the reduction was seen in the majority of cases and we never observed an increase of laccase activity in EP155/EP713, our results on this system go in line with previous research [11,13,14]. Interestingly, we did not observe the same consistent LAC activity reduction with the other virus strains utilized in our study. Rather, as mentioned before, we frequently observed an increase in LAC activity, which was especially evident on solid media. Even more surprisingly, the same prototypic viral strain EP713, when introduced into fungal isolates other than EP155, showed exactly the opposite effect by increasing LAC activity (e.g., on MEA in EP155/EP713 we observed a 100% smLAC activity reduction, while in L14/EP713, D7/EP713 and D9/EP713 there was an increase of 19.6%, 21% and 940% of smLAC activity when compared to the isogenic virulent fungal counterpart, respectively). If we focus only on ecLAC, considering its putative role in the pathogenesis of chestnut blight, we see that the majority of fungus/virus combinations showed the same reductive effect as observed in EP155/EP713, but in fungal isolate D7 all of the introduced virus strains increased ecLAC activity, though not always significantly. It has been speculated that CHV1′s reductive effect on *C. parasitica* laccase is an important contributing factor for the reduction of fungal virulence [4]. Similar effects have been shown in phytopathogenic fungi *Diaporthe ambigua*, *B. cinerea* and *Sclerotium rolfsii* where mycoviruses have led to a hypovirulent effect and reduced LAC activity [42,43,44]. Interestingly, *Cryphonectria hypovirus 3* also causes hypovirulence in *C. parasitica*, but without significant LAC reduction [45], while in hypovirulent *B. cinerea* infected with *Botrytis cinerea mitovirus 1* increased LAC activity was reported [46]. In *Fusarium circinatum*, the presence of a virus from the *Narnaviridae* family led to an increased virulence of the fungus without any effect on the fungal LAC [47]. Clearly, the laccases of different fungi respond to various mycoviral infections differently. However, we have demonstrated that isolates of the same fungal species, except differing among themselves in laccase activity, have strikingly different responses to a CHV1 infection. As shown previously for stress enzymes [20] and fungal genome methylation levels [34], the effect of CHV1 on laccase activity is not solely determined by the virus strain, but by a particular fungal isolate and their specific combinations.

## 5. Conclusions

Considering the important role of CHV1 in chestnut blight biocontrol, every aspect of a virus’ effect on the metabolism of a fungus should be studied in detail. Thus, having in mind the diversity of *C. parasitica* and CHV1 in natural populations, this work demonstrated the metabolic variability of *C. parasitica* isolates and the effect of different virus strains on the laccase activities of several fungal hosts. We have shown that some CHV1 strains increase the activity of *C. parasitica* laccases of certain fungal isolates upon infection, which is contrary to previous assumptions, and that the effect is strongly influenced by culture conditions and fungal/viral genotypes. This stresses the importance of conducting this kind of enzymatic activity assay on genetically diverse host isolates and biocontrol agent strains to better reflect their natural phenotypic variability and potentially foresee plausibly different effects of host/virus combinations. Our work demonstrated the importance of conducting this type of study in experimental systems and culture conditions that resemble natural conditions as much as possible. As the habitat of *C. parasitica* is chestnut bark, it would be advisable to conduct this type of research on solid media to be able to infer the most relevant conclusions applicable to natural populations.

## Figures and Tables

**Table 1:** The transfected *Cryphonectria parasitica* cultures obtained by transferring CHV1 strains from virus–donor *C. parasitica* isolates into virulent fungal isolates. Virulent fungal isolates were used as virus-free controls, thus giving a total of 17 experimental cultures.

		Virulent Fungal Isolates			
		EP155	L14	D7	D9
Virus- Donor	EP713	EP155/EP713	L14/EP713	D7/EP713	D9/EP713
	CR23	-	L14/CR23	D7/CR23	D9/CR23
	HK27	-	L14/HK27	D7/HK27	D9/HK27
	SHE30	-	L14/SHE30	D7/SHE30	D9/SHE30

**Table 2:** Main effects ANOVA of laccase activity of *Cryphonectria parasitica* isolates infected with different *Cryphonectria hypovirus 1* strains or uninfected controls, when grown on different growth media for 5 or 10 days. Statistically significant differences at *p* < 0.05 are denoted by an asterisk (*).

		SOLID MEDIA			LIQUID MEDIA					
		smLAC			icLAC			ecLAC		
Source	Df ^1^	MS ^2^	F ^3^	*p*	MS	F	*p*	MS	F	*p*
DURATION ^a^	1	102.694	8.9929	0.003 *	9.90002	14.3188	0.0002 *	0.005404	0.04547	0.8314
MEDIUM RICHNESS ^b^	1	1265.916	110.8561	<0.0001 *	10.95784	15.8487	0.0001 *	0.350012	2.94490	0.0877
FUNGUS ^c^	3	65.347	5.7224	0.0009 *	2.96914	4.2944	0.006 *	0.230932	1.94300	0.1240
VIRUS ^d^	4	126.814	11.1051	<0.0001 *	3.54686	5.1300	0.0006 *	1.322938	11.13082	<0.0001 *
Error	194	11.419			0.69140			0.118854		

^1^ degrees of freedom, ^2^ mean square value, ^3^ F ratio; NOTE: The following effects of the factors were tested: ^a^ duration of cultivation (five or ten days), ^b^ nutritional value of the growth medium (potato dextrose agar (PDA) or broth (PDB) were considered rich, while malt extract (ME) or malt extract agar (MEA) were considered poor), ^c^ fungal isolate and ^d^ virus strain on laccase activities of fungi grown either on solid media (PDA or MEA) or liquid media (PDB or ME). On solid media, laccase activity was defined as the sum of all intracellular and majority of extracellular laccase and designated smLAC, while on liquid media intracellular (icLAC) and extracellular (ecLAC) laccase activities were analyzed separately. Laccase activity is expressed as nmol of generated DMOPox per minute per mg of mycelium dry weight.

**Table 3:** The laccase activities of four virus-free fungal isolates after 10 days of growth on different media, that is, solid potato dextrose agar (PDA) and malt extract agar (MEA), liquid potato dextrose broth (PDB) and malt extract (ME). Laccase activity is expressed as nmol of generated DMOPox per minute per mg of mycelium dry weight. Data represent the mean of three replicates ± standard error. Letters denote statistically significant differences between values in the same column, that is, fungal isolates grown on the same medium, at *p* < 0.05.

FUNGAL ISOLATE	PDA smLAC ^1^	MEA smLAC	PDB		ME	
			icLAC ^2^	ecLAC ^3^	icLAC	ecLAC
EP155	0.00 ± 0.00 ^b^	0.16 ± 0.11 ^a^	0.59 ± 0.14 ^b^	0.40 ± 0.17 ^b^	0.96 ± 0.06 ^a^	0.18 ± 0.03 ^a^
L14	0.36 ± 0.03 ^a^	0.00 ± 0.00 ^a^	1.29 ± 0.08 ^a^	1.46 ± 0.25 ^a^	1.00 ± 0.04 ^a^	0.00 ± 0.00 ^b^
D7	0.29 ± 0.14 ^ab^	0.00 ± 0.00 ^a^	0.51 ± 0.09 ^b^	0.09 ± 0.01 ^b^	0.77 ± 0.11 ^a^	0.00 ± 0.00 ^b^
D9	0.02 ± 0.01 ^b^	0.02 ± 0.01 ^a^	0.87 ± 0.14 ^ab^	0.66 ± 0.16 ^b^	1.02 ± 0.04 ^a^	0.16 ± 0.01 ^a^

^1^ laccase activity on solid media, ^2^ intracellular laccase activity, ^3^ extracellular laccase activity.

## Data Availability

All data obtained in this research is available upon request.
